# The association between intrahepatic malignant tumors and hepatitis B-related pathological cirrhosis: a retrospective cohort study

**DOI:** 10.3389/fmed.2025.1618715

**Published:** 2025-12-09

**Authors:** Fengping Li, Xiaofei Liu, Wei Lu, Dan Huang, Weijia Lin, Zhanqing Zhang

**Affiliations:** Shanghai Public Health Clinical Center Affiliated to Fudan University, Shanghai, China

**Keywords:** hepatocellular carcinoma, hepatitis B virus (HBV), liver fibrosis, cirrhosis, intrahepatic malignant tumor, predictive model, predictive factors

## Abstract

**Background:**

Hepatocellular carcinoma (HCC) is one of the most common and aggressive malignancies worldwide, with chronic hepatitis B virus (HBV) infection being the primary etiology. HBV-induced liver fibrosis and cirrhosis are significant pathological foundations for the development of HCC. Although several predictive models for HCC in patients with chronic hepatitis B (CHB) exist, a unified model for predicting the progression from cirrhosis, based on pathological diagnosis, to HCC has not yet been established. This study aims to explore the probability and predictive factors of intrahepatic malignant tumor development from a pathological perspective, providing a theoretical basis for clinical intervention.

**Patients and methods:**

This retrospective study enrolled patients with HBeAg-positive CHB who had pathological cirrhosis (Scheuer/Ludwig stage S4) at the Shanghai Public Health Clinical Center before April 2023. Inclusion criteria comprised persistent HBsAg positivity for at least 6 months and pathological cirrhosis (stage S4) with disease remission following antiviral therapy. Exclusion criteria included cirrhosis stages 0–3, concurrent infections with other viruses, and severe comorbidities. A total of 471 patients were included, with 34 developing HCC during follow-up. Patients were randomly assigned to a training set (*n* = 328) and a validation set (*n* = 143). Univariate and stepwise multivariate logistic regression analyses were performed to identify independent risk factors for HCC, and a predictive nomogram was constructed. The model's performance was evaluated using the area under the receiver operating characteristic curve (AUC), concordance index (C index), calibration curves, and decision curve analysis (DCA).

**Results:**

The incidence of HCC was 4.89% in the training set and 2.34% in the validation set. Univariate analysis identified age, CHE, WBC, Hb, PLT, ANC, AMC, HA, and CIV as significantly associated with HCC development. Multivariate analysis confirmed age, WBC, C4, and CIV as independent predictive factors. The nomogram based on these factors demonstrated satisfactory predictive performance, with AUC values of 0.869 and 0.762 in the training and validation sets, respectively. Calibration curves showed good agreement between predicted and actual outcomes in both sets. Decision curve analysis indicated that the model's net benefit was significantly higher than that of “treat-all” or “treat-none” strategies when the high-risk threshold was set between 5% and 40%, highlighting its clinical utility.

**Conclusion:**

This study developed a predictive model for HCC based on age, WBC, C4, and CIV in patients with HBV-related cirrhosis. The model effectively predicted the risk of HCC and provided a reference for clinical intervention. Despite limitations in sample size, the model exhibited robust predictive performance and clinical applicability. Future work should validate the model in multicenter studies and integrate multi-omics data to develop a more comprehensive predictive system.

## Introduction

Hepatocellular carcinoma (HCC) is one of the most aggressive malignancies, ranking as the second leading cause of cancer-related death worldwide (1, 2). Chronic hepatitis B virus (HBV) infection remains the dominant etiology of HCC, accounting for approximately 50%−80% of cases in high-incidence regions (3). Globally, an estimated 254 million people were chronically infected with HBV in 2022; the African, Western Pacific and South-East Asia regions together account for approximately 88% of this burden (4). To date, hundreds of HBV genomic sequences have been reported and, based on geographical differences (5, 6), classified into 10 genotypes (A–J) (7–9). Genotype A, for example, prevails in Western Europe, the United States, Central Africa and India (10), whereas genotypes B and C are found mainly in East Asia (including China, Japan and South Korea) (11–13). Genotype D is distributed in Central Asia, Africa, the Mediterranean region and India (14), and genotype E is restricted to West Africa (15). Genotypes F–I together account for less than 2% of global HBV infections (6, 16). Countries with large immigrant populations are more likely to show coexistence of multiple HBV genotypes (17). In China, genotypes B and C jointly dominate, with a combined prevalence of approximately 90% (5, 18). Consequently, among individuals with chronic HBV infection who are untreated, 15%−40% progress to cirrhosis, which may lead to liver failure and liver cancer (19). The Global Burden of Disease Study 2021 estimated that in a single year HBV caused approximately 1.2 million incident cases of cirrhosis and 288,000 new cases of primary liver cancer, leading to about 970,000 liver-related deaths—a mortality toll that exceeds that of malaria and is comparable to that of tuberculosis (20). In China, recent Global Burden of Disease estimates indicate that HBV accounts for approximately 68% of cirrhosis cases and 65% of hepatocellular carcinoma cases, making it the leading reversible cause of both diseases (21). These epidemiological data frame HBV-driven cirrhosis and HCC as urgent public-health problems that require targeted prevention strategies.

The sequence from persistent HBV infection to HCC is orchestrated through cycles of hepatocyte necrosis, inflammation and regeneration. Viral proteins and the host immune response activate Kupffer cells and hepatic stellate cells (HSCs), triggering fibrogenesis. Activated HSCs differentiate into myofibroblasts that deposit excess extracellular matrix (ECM), reducing sinusoidal perfusion and gradually replacing functional parenchyma with scar tissue (22–24). Histologically, advanced fibrosis (cirrhosis) is characterized by fibrous septa that link portal tracts to central veins, encapsulating regenerative nodules and distorting intrahepatic blood flow. The resulting portal hypertension and hepatocellular dysfunction create a micro-environment in which genetically altered hepatocytes undergo clonal expansion, yielding dysplastic foci that may ultimately progress to HCC (25, 26). Accordingly, 80%−90% of HCCs arise in cirrhotic livers, and approximately one-third of patients with cirrhosis will develop HCC during follow-up (27).

Although a variety of scores [REACH-B (28), PAGE-B (29), mPAGE-B (30), etc.] estimate HCC risk in CHB cohorts, no unified model specifically predicts malignant transformation once pathologically confirmed HBV-related cirrhosis has been established. Filling this gap would enable clinicians to stratify patients for intensified surveillance or adjuvant therapy, potentially improving outcomes in a setting where the 5-year survival of advanced HCC remains < 12% (3). Therefore, the present study aims to quantify the probability and to identify independent predictors of intrahepatic malignant tumors in patients with a pathological diagnosis of HBV-related cirrhosis, thereby providing an evidence base for targeted tumor prevention in this high-risk population.

## Patients and methods

### Patients

Before April 2023, consecutive patients with chronic hepatitis B (CHB) from our institution (Shanghai Public Health Clinical Center Affiliated to Fudan University) were retrospectively enrolled in this study and randomly assigned to training and validation sets in a 7:3 ratio using a computer-generated random sequence, stratified by HCC outcome to ensure balanced distribution of events across sets.

The inclusion criteria were as follows:

1. HBeAg-positive chronic HBV infection with pathological diagnosis of cirrhosis (All patients were HBeAg-positive at the time of initial diagnosis of cirrhosis. Disease remission was achieved following antiviral therapy, regardless of subsequent HBeAg seroconversion status);
2. A confirmed diagnosis of chronic active hepatitis B, defined as persistent positivity for HBsAg for at least 6 months, with negative HBsAb;
3. Pathological diagnosis of cirrhosis (pathological stage S4), with either a tendency toward spontaneous remission of hepatitis activity or remission following oral antiviral therapy (OAT), and who had undergone two or more pathological assessments.

The exclusion criteria were as follows:

1. Patients with cirrhosis graded as stages 0–3;
2. Allergy to interferon or antiviral medications;
3. Concurrent infection with other viruses, including HIV, HCV, HAV, HDV, or HEV;
4. Presence of other liver diseases, such as fatty liver disease, alcoholic liver disease, toxic liver disease, hereditary liver disease, or gallbladder or biliary tract stones;
5. History of severe diseases in vital organs or tissues (e.g., brain, heart, and kidneys);
6. Patients deemed unsuitable for inclusion after assessment.

The cutoff date for data collection in this study was April 30, 2023. Demographic, laboratory, and pathological data for each patient were retrospectively collected from the hospital information system and laboratory information system of our institution. The follow-up start date was defined as the date of the first visit or the first liver biopsy, and the endpoint was the date of the last follow-up before the cutoff date for data collection.

The study was conducted in accordance with the Declaration of Helsinki and approved by the Institutional Ethics Committee of the Shanghai Public Health Clinical Center Affiliated to Fudan University. Informed consent was obtained from all patients for their data to be used for research.

### Pathological diagnosis

The liver pathological diagnostic criteria employed in this study were based on the Scheuer/Ludwig scoring system (31). The histological grading of inflammation activity ranges from G0 to G4, while the histological staging of fibrosis ranges from S0 to S4 (see Table 1).

**Table 1:** Scheuer scoring system.

**Fibrosis and cirrhosis staging**	**Grading of inflammatory and necrotic activity in chronic hepatitis**	**Score**
Portal/periportal activity	None or minimal	0
	Portal inflammation only	1
	Mild interface hepatitis	2
	Moderate interface hepatitis	3
	Severe interface hepatitis	4
Lobular activity	None	0
	Inflammatory cells but no hepatocellular death	1
	Focal cell death	2
	Severe focal cell death, with or without confluent necrosis	3
	Damage including bridging necrosis	4
Fibrosis	None	0
	Enlarged, fibrotic portal tracts	1
	Periportal or portal-portal septa but intact architecture	2
	Fibrosis with architectural distortion but no obvious cirrhosis	3
	Probable or definite cirrhosis	4

According to the Scheuer/Ludwig criteria, we defined the initial biopsy showing a histological stage of S4 as cirrhosis. All liver tissue samples were obtained via ultrasound-guided percutaneous aspiration liver biopsy using a 16G biopsy needle. Immediately after collection, the tissue specimens were placed into pre-prepared cryovials and transported for examination under low-temperature conditions. The liver tissue samples were processed within 36 h by dedicated pathologists in Shanghai Public Health Clinical Center. The quality assessment and pathological diagnosis were independently conducted by a senior pathologist.

HCC was diagnosed according to the American Association for the Study of Liver Diseases (AASLD) criteria—non-invasive imaging showing arterial hyperenhancement with wash-out on multiphasic CT/MRI, or, if imaging was atypical, pathological confirmation and surveillance imaging was scheduled every 6 months. No central imaging review was conducted, instead, two radiologists with ≥10 years of hepatobiliary imaging experience in Shanghai Public Health Clinical Center independently interpreted each study and reached consensus.

### Data-extraction protocol

The clinical data and laboratory results were retrospectively extracted from the Electrical Medical Record System of the institution. The alanine aminotransferase (ALT), aspartate aminotransferase (AST), alkaline phosphatase (ALP), gamma-glutamyl transferase (GGT), cholinesterase (CHE), serum direct bilirubin (SDB), serum albumin (Alb), serum globulin (GLB) albumin–globulin ratio (A/G), prealbumin (PA), international normalized ratio (INR), white blood cells (WBC), red blood cells (RBC), hemoglobin (Hb), platelets (PLT), absolute neutrophil count (ANC), absolute lymphocyte count (ALC), absolute monocyte count (AMC), immunoglobulin A (IgA), immunoglobulin G (IgG), immunoglobulin M (IgM), complement component 3 (C3), complement component 4 (C4), hepatitis B surface antigen (HBsAg), hepatitis B e antigen (HBeAg), hepatitis B virus DNA (HBVDNA), alpha-fetoprotein (AFP), hyaluronic acid (HA), procollagen type III (PIIIP), and collagen type IV (CIV) were extracted from the laboratory tests.

### Statistical analysis

Among the included patients, those who developed HCC were grouped as HCC, whereas the others were grouped as non-HCC. Statistical algorithms were used to verify the concordance between the two groups. Data distribution was evaluated using the Kolmogorov–Smirnov test. Continuous variables are presented as mean ± standard deviation (SD) if normally distributed, whereas the non-normally distributed variables are viewed as median (first-third quartile value). Categorical variables are listed as numbers (percentages). Statistical differences for categorical, continuous, and layered variables were examined using Pearson's chi-square, Student's *t*-test, and rank-sum test, respectively.

The significance of each variable in the training cohort was assessed by univariate logistic regression analysis for investigating the independent risk factors of presence of HCC. All variables associated with HCC at a significant level were candidates for stepwise multivariate analysis. This study follows the Transparent Reporting of a multivariable prediction model for Individual Prognosis OR Diagnosis (TRIPOD) guideline.

A nomogram was formulated based on the results of multivariate logistic regression analysis and by using the rms package of R, version 4.4.2. The nomogram is based on proportionally converting each regression coefficient in multivariate logistic regression to a 0- to 100-point scale. The effect of the variable with the highest β coefficient (absolute value) is assigned 100 points. The points are added across independent variables to derive total points, which are converted to predicted probabilities. The predictive performance of the nomogram was measured by concordance index (C index) and calibration with 1,000 bootstrap samples to decrease the overfit bias (32).

For clinical use of the model, the total scores of each patient were calculated based on the nomogram. Predictive models for HCC were formulated based on a multivariate-regression model and then used to compute HCC risk scores for each patient. The area under the curve (AUC) was adopted to assess the model's performance. An AUC of 0.5–0.7, 0.7–0.9, and >0.9 would be considered modest, good, and excellent, respectively. The Youden index was calculated to determine an ideal threshold for high risk of HCC. Also, constructing decision curve analysis (DCA) curves to evaluate the clinical utility of the model.

In all analyses, *P* < 0.05 was considered to indicate statistical significance. All analyses were performed using R software (Version 4.4.2). Missing data for the analyzed variables were minimal (< 5% for any variable). Therefore, a complete-case analysis was performed, wherein only patients with complete data for all variables of interest were included in the logistic regression modeling.

## Results

### Clinicopathologic characteristics

**Figure 1:**
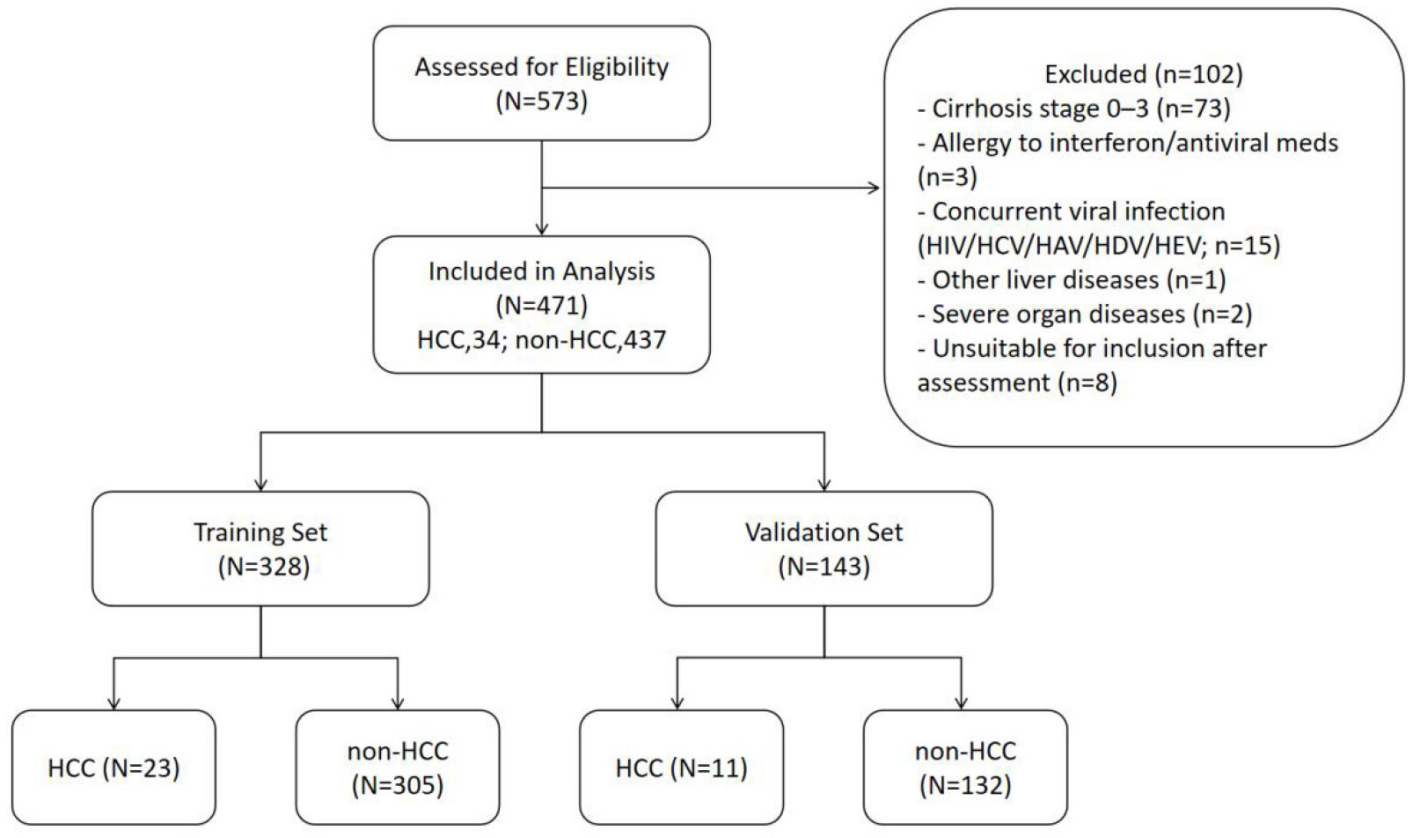
Elements of patients enrolled in this study. Five hundred seventy-three patients with HBeAg-positive chronic HBV infection and pathological diagnosis of cirrhosis (pathological stage S4) before 2023 were enrolled in this study; among them, 102 were excluded according to the exclusion criteria. The remaining 471 were randomly assigned to the training set (328) and validation set (143). There were 34 cases (7.22%) of HCC in the included patients. HCC, hepatocellular carcinoma; non-HCC, non hepatocellular carcinoma.

A total of 573 patients with HBeAg-positive chronic HBV infection and pathological diagnosis of cirrhosis (pathological stage S4) were followed up regularly at the Shanghai Public Health Clinical Center. For criteria selection, 102 were excluded and 471 satisfied the inclusion criteria, of whom 34 developed hepatocellular carcinoma during the follow-up period, while 437 did not (Figure 1). Among them, 328 patients were randomly assigned to the training group. The rest were validated. The total liver cancer incidence rate was 7.22%, with 4.89% in the training group and 2.34% in the validation group. The median followup time was 95.04 (range, 29.11–125.60) months and 95.43 (range, 29.50–122.49) months in the training and validation cohorts, respectively.

**Figure 2:**
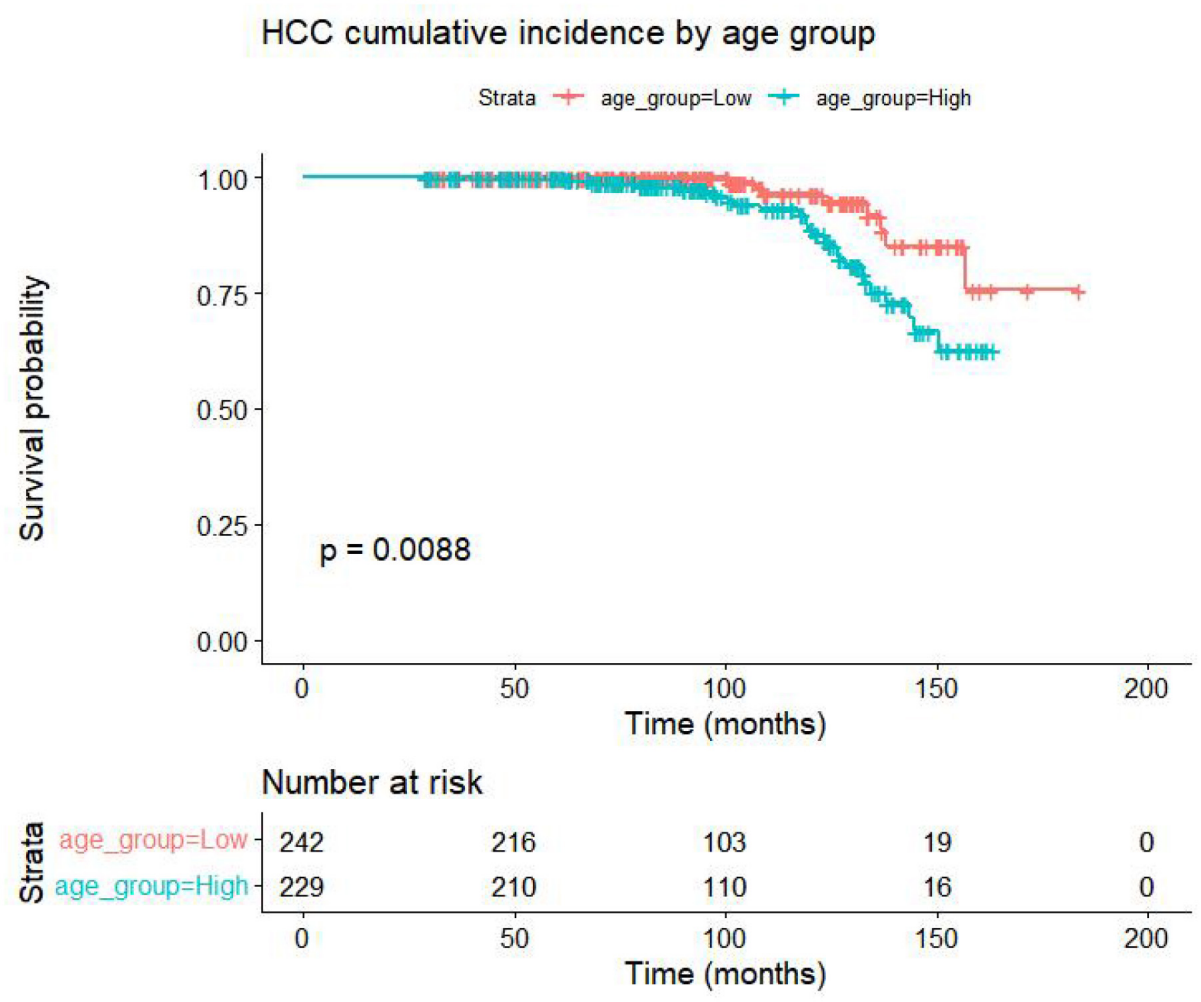
Kaplan–Meier estimates of cumulative HCC incidence by age group in patients with biopsy-proven HBV-related cirrhosis (Log-rank *P* = 0.009). Patients were stratified by the median age (47 years) into Low ( ≤ 47 years, *n* = 242) and High (>47 years, *n* = 229) groups. Follow-up was censored on 30 April 2023. The *y*-axis shows the cumulative incidence of HCC; the *x*-axis shows follow-up time (months). The risk table (bottom right) indicates the number of patients still at risk at each time point.

The baseline characteristics of the patients are listed in Table 2. The baseline clinicopathologic data were similar between the training and validation cohorts. As detailed in Table 2, a markedly higher proportion of patients in the HCC group were of advanced age (mean age 53.4 vs. 41.3 years). Also, Kaplan–Meier curves stratified by median age ( ≤ 47 vs. >47 years) confirmed a higher cumulative incidence in older patients (Log-rank *P* = 0.009, Figure 2). Notably, the prevalence of a low white blood cell count (WBC ≤ 4.5 × 10^9^/L) was 78.3% (18/23) in the HCC group compared to only 41.6% (126/303) in the non-HCC group. Similarly, a low complement C4 level (C4 ≤ 0.15 g/L) was present in 87.0% (20/23) of HCC patients vs. 61.8% (183/296) of non-HCC patients. For the fibrosis markers, a high hyaluronic acid level (HA >120 ng/ml) was observed in 87.0% (20/23) of HCC cases, compared to 58.2% (174/299) of non-cases. Conversely, a low type IV collagen level (CIV ≤ 75 ng/ml) was associated with HCC, found in 91.3% (21/23) of the HCC group and 67.2% (201/299) of the non-HCC group. These stark contrasts in the distribution of age, WBC, C4, HA, and CIV between the two groups underscore their strong association with the development of HCC in this cohort of patients with cirrhosis.

**Table 2:** Patient characteristics in the training and validation sets.

**Variable**	**Training set**				**Validation set**				*P* ^*^
	**Total (328)**	**Non-HCC (305)**	**HCC (23)**	***P***	**Total (143)**	**Non-HCC (132)**	**HCC (11)**	***P***	
**HBsAg (%),COI**									
HBsAg ≤ 4,000	214 (66.9)	195 (65.4)	19 (86.4)	0.075	98 (68.5)	91 (68.9)	7 (63.6)	0.979	0.807
15.6-2.2,-1.3498ptHBsAg > 4,000	106 (33.1)	103 (34.6)	3 (13.6)		45 (31.5)	41 (31.1)	4 (36.4)		
**HBsAglog (%)**									
HBsAglog ≤ 3	96 (30.0)	86 (28.9)	10 (45.5)	0.162	32 (22.4)	30 (22.7)	2 (18.2)	1.000	0.114
15.6-2.2,-1.3498ptHBsAglog > 3	224 (70.0)	212 (71.1)	12 (54.5)		111 (77.6)	102 (77.3)	9 (81.8)		
**HBeAg (%), COI**									
HBeAg ≤ 200	241 (76.3)	223 (76.1)	18 (78.3)	1.000	103 (73.0)	95 (72.5)	8 (80.0)	0.885	0.536
15.6-2.2,-1.3498ptHBeAg > 200	75 (23.7)	70 (23.9)	5 (21.7)		38 (27.0)	36 (27.5)	2 (20.0)		
**HBeAglog (%)**									
HBeAglog ≤ 2	102 (51.5)	98 (52.4)	4 (36.4)	0.469	45 (51.1)	40 (49.4)	5 (71.4)	0.468	1.000
15.6-2.2,-1.3498ptHBeAglog > 2	96 (48.5)	89 (47.6)	7 (63.6)		43 (48.9)	41 (50.6)	2 (28.6)		
**HBeAg (%)**									
Negative	127 (38.7)	115 (37.7)	12 (52.2)	0.249	53 (37.1)	50 (37.9)	3 (27.3)	0.708	0.813
15.6-2.2,-1.3498ptPositive	201 (61.3)	190 (62.3)	11 (47.8)		90 (62.9)	82 (62.1)	8 (72.7)		
**HBVDNA (%), IU/ml**									
HBVDNA ≤ 100,000	150 (45.7)	141 (46.2)	9 (39.1)	0.658	34 (23.8)	32 (24.2)	2 (18.2)	0.932	< 0.001
HBVDNA > 100,000	178 (54.3)	164 (53.8)	14 (60.9)		109 (76.2)	100 (75.8)	9 (81.8)		
**AFP (%), ng/ml**									
AFP < 20	180 (56.6)	166 (56.3)	14 (60.9)	0.850	74 (53.2)	67 (52.3)	7 (63.6)	0.609	0.515
20 ≤ AFP ≤ 400	114 (35.8)	107 (36.3)	7 (30.4)		00 57 (41.0)	53 (41.4)	4 (36.4)		
15.6-2.2,-1.3498ptAFP > 400	24 (7.5)	22 (7.5)	2 (8.7)		8 (5.8)	8 (6.2)	0 (0.0)		
**HA (%), ng/ml**									
HA ≤ 120	128 (39.8)	125 (41.8)	3 (13.0)	0.013	63 (44.7)	59 (45.4)	4 (36.4)	0.793	0.374
15.6-2.2,-1.3498ptHA > 120	194 (60.2)	174 (58.2)	20 (87.0)		78 (55.3)	71 (54.6)	7 (63.6)		
**PIIIP (%), ng/ml**									
PIIIP ≤ 82	234 (72.7)	213 (71.2)	21 (91.3)	0.066	96 (68.1)	86 (66.2)	10 (90.9)	0.176	0.372
15.6-2.2,-1.3498ptPIIIP > 82	88 (27.3)	86 (28.8)	2 (8.7)		45 (31.9)	44 (33.8)	1 (9.1)		
**CIV (%), ng/ml**									
CIV ≤ 75	222 (68.9)	201 (67.2)	21 (91.3)	0.030	94 (66.7)	84 (64.6)	10 (90.9)	0.149	0.707
CIV > 75	100 (31.1)	98 (32.8)	2 (8.7)		47 (33.3)	46 (35.4)	1 (9.1)		

Baseline information and serum tests were statistically analyzed. P represents the statistical significance between the HCC and non-HCC groups within each set. P^*^ represents the statistical significance between the training set and validation set. Continuous variables are presented as mean±standard deviation (SD) if normally distributed, whereas non-normally distributed variables are shown as median (first-third quartile value). Categorical variables are listed as numbers (percentages).

OAT, oral anti-viral treatment.

### Development and validation of a predictive nomogram

**Figure 3:**
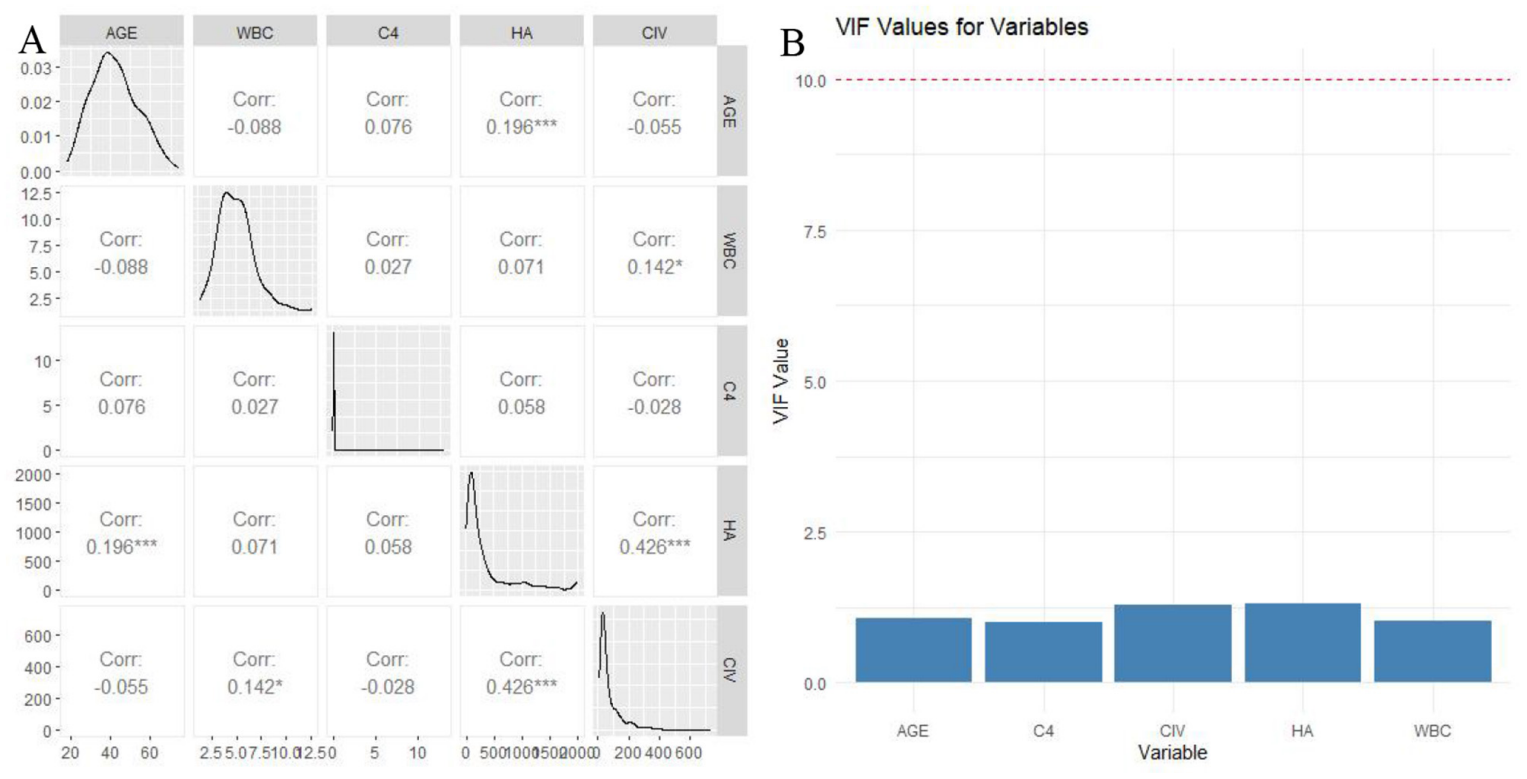
Correlation analysis **(A)** and multicollinearity diagnostics **(B)** among Age, C4, CIV, HA and WBC.

In the training set, univariable logistic regression was performed (Table 3), in which Age, CHE, WBC, Hb, PLT, ANC, AMC, HA, and CIV was statistically significant. We further conducted stepwise regression analysis to select variables based on the results of univariate logistic analysis, ultimately identifying five variables: age, WBC, C4, HA, and CIV (Table 4). Correlation analysis and multicollinearity diagnostics among these five variables showed no significant correlation or multicollinearity (Figure 3).

**Table 3:** Univariate logistic-regression analysis of HCC based on population characters in the training set.

**Variable**	**OR**	**Lower CI**	**Upper CI**	***P-*value**
Age	1.103	1.059	1.153	**< 0.0001**
Sex, male vs. female	1.689	0.613	5.957	0.354
OAT, yes vs. no	3.67	1.044	23.274	0.084
OAT course	1.027	0.984	1.07	0.212
ALT, >40 vs. ≤ 40	0.892	0.356	2.546	0.816
AST, >35 vs. ≤ 35	1.576	0.571	5.565	0.421
ALP, >110 vs. ≤ 110	2.004	0.855	4.835	0.111
GGT, >64 vs. ≤ 64	0.855	0.364	2.028	0.717
CHE, >5,680 vs. ≤ 5,680	0.358	0.126	0.887	**0.035**
SDB, >7 vs. ≤ 7	1.14	0.485	2.813	0.767
Alb, >40 vs. ≤ 40	0.573	0.215	1.385	0.234
GLB, >40 vs. ≤ 40	2.581	0.705	7.585	0.109
A/G, >1.7 vs. ≤ 1.7	0.285	0.016	1.413	0.225
PA, >35 vs. ≤ 35	0.681	0.216	3.017	0.555
TBA, >10 vs. ≤ 10	2.321	0.769	10.043	0.183
INR, >1.2 vs. ≤ 1.2	1.808	0.768	4.301	0.173
RBC	0.265	0.122	0.549	**< 0.0001**
WBC, >4.5 vs. ≤ 4.5	0.198	0.064	0.51	**0.002**
Hb, >130 vs. ≤ 130	0.295	0.122	0.696	**0.006**
PLT, >110 vs. ≤ 110	0.174	0.049	0.475	**0.002**
ANC, >2.7 vs. ≤ 2.7	0.111	0.018	0.387	**0.003**
ALC, >1.7 vs. ≤ 1.7	0.406	0.143	1.006	0.065
AMC, >0.3 vs. ≤ 0.3	0.283	0.106	0.684	**0.007**
IgA, >3 vs. ≤ 3	2.413	0.999	6.434	0.060
IgG, >15 vs. ≤ 15	2.1	0.846	5.958	0.129
IgM, >2 vs. ≤ 2	0.656	0.268	1.767	0.374
C3, >0.8 vs. ≤ 0.8	0.555	0.196	1.381	0.230
C4, >0.15 vs. ≤ 0.15	0.243	0.056	0.729	**0.025**
HBsAg, >4,000 vs. ≤ 4,000	0.299	0.069	0.903	0.056
HBsAglog, >3 vs. ≤ 3	0.487	0.202	1.193	0.107
HBeAg, >200 vs. ≤ 200	0.885	0.284	2.311	0.815
HBeAglog, >2 vs. ≤ 2	1.927	0.563	7.561	0.308
HBeAg, yes vs. no	0.555	0.234	1.306	0.174
HBVDNA, >100,000 vs. ≤ 100,000	1.337	0.569	3.298	0.511
AFP				0.596
≥20 and ≤ 400 vs. < 20	0.776	0.286	1.928	0.596
>400 vs. < 20	1.078	0.162	4.211	0.924
HA, >120 vs. ≤ 120	4.789	1.598	20.641	**0.013**
PIIIP, >80 vs. ≤ 80	0.236	0.037	0.828	0.054
CIV, >75 vs. ≤ 75	0.195	0.031	0.684	**0.029**

OR, odds ratio. Bold values indicate statistically significant differences.

**Table 4:** Multivariate logistic regression analysis of HCC based on population characters in the training set.

**Variable**	**Multivariate logistic regression analysis**		
	**β^a^**	**OR (95% CI)**	***P*-value**
Age	0.086	1.090 (1.041–1.146)	< 0.001
WBC, >4.5 vs. ≤ 4.5	−1.230	0.292 (0.087–0.836)	0.030
C4, >0.15 vs. ≤ 0.15	−1.477	0.228 (0.486–0.774)	0.031
HA, >120 vs. ≤ 120	1.169	3.217 (0.943–14.821)	0.086
CIV, >75 vs. ≤ 75	−1.862	0.155 (0.023–0.600)	0.018

OR, odds ratio.

^a^Unstandardized β coeficients were calculated from the multivariate logistic regression model.

We then constructed a multivariate model using these five variables. The results revealed that Age, WBC, C4, and CIV were statistically significant. The weight of each parameter was determined by regression coefficients in the multivariable model (Table 2). The risk-score formula was as follows:

Logit (*P*) = −6.329 + 0.086 × age – 1.230 × *I* (WBC >4.5) – 1.477 × *I* (C4 >0.15) – 1.862 × *I* (CIV > 75).

**Figure 4:**
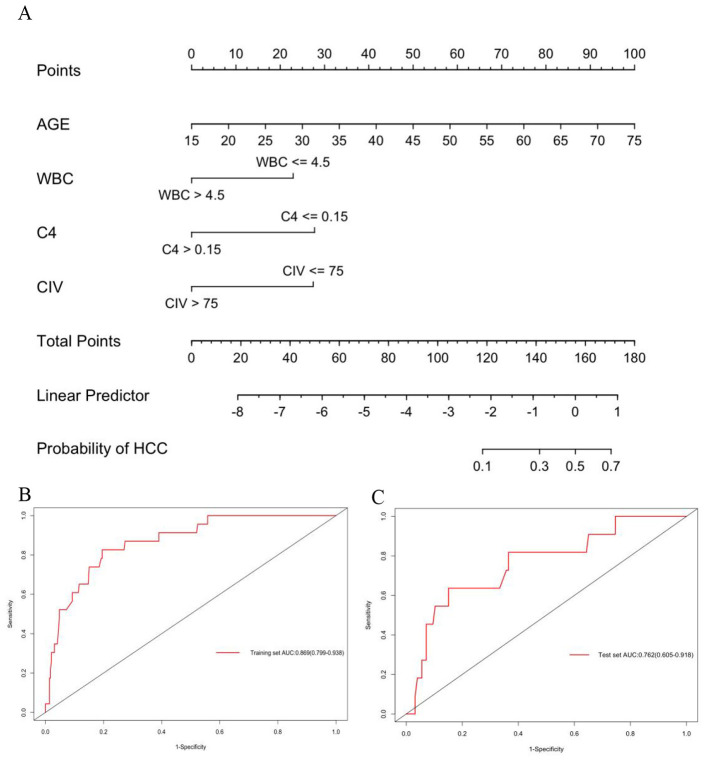
Predictive nomogram for HCC risk and its predictive performance. **(A)** Nomogram to estimate the risk of HBeAg positive CHB who had pathological cirrhosis. To use the nomogram, find the position of each variable on the corresponding axis, draw a line to the points axis for the number of points, add the points from all of the variables, and draw a line from the total points axis to determine the HCC probabilities at the lower line of the nomogram; **(B, C)** Internal and external AUC were calculated to evaluate the performance of this model. The AUC was comparable in both sets and showed outstanding precision.

For each patient, the HCC risk scores were calculated. After that, ROC curves were plotted for internal (training wing) and external validation (test wing) to verify the model's performance (Figures 4B, C). The AUC was 0.869 [95% confidence interval (CI), 0.799–0.938] and 0.762 (95% CI, 0.605–0.918) in the training and validation sets, respectively, indicating satisfactory performance. In the training set, the Youden value peaks with the threshold of HCC risk at 0.631.

To aid its bedside utility, we used a nomogram to visualize this model. The nomogram and prediction performance via ROC curves in the internal and external validation are shown in Figure 4A.

### Time-to-event validation of predictive factors

To further verify the robustness of the four variables included in the nomogram, we conducted a Cox analysis for time-to-event validation. Univariate Cox analysis showed that each 1-year increase in age was associated with a 5% increase in HCC incidence (HR = 1.05, 95% CI 1.02–1.08, *P* < 0.001), while low WBC remained significantly protective (HR = 0.32, 95% CI 0.15–0.69, *P* = 0.004; Table 5).

**Table 5:** Univariate and multivariate Cox proportional hazards analysis of HCC occurrence.

**Variable**	**HR (95% CI)**	***P*-value**	**Adjusted HR (95% CI)**	***P*-value**
15.6-2.2,-1.3498ptAge (per 1 year)	1.050 (1.020–1.080)	0.0004	1.050 (1.020–1.080)	0.002
**WBC**				
Low ( ≤ 4.5^*^10^9^/L)	1 (Ref.)			
15.6-2.2,-1.3498ptHigh (>4.5^*^10^9^/L)	0.324 (0.151–0.694)	0.004	0.377 (0.174–0.817)	0.014
**C4**				
Low ( ≤ 0.15g/L)	1 (Ref.)			
15.6-2.2,-1.3498ptHigh (>0.15g/L)	0.871 (0.416–1.820)	0.715	1.090 (0.517–2.310)	0.817
**CIV**				
Low ( ≤ 75ng/ml)	1 (Ref.)			
High (>75ng/ml)	0.412 (0.158–1.075)	0.070	0.523 (0.158–1.730)	0.287

After simultaneous adjustment in multivariate Cox model, age (HR = 1.05, 95% CI 1.02–1.08, *P* = 0.002) and low WBC (HR = 0.38, 95% CI 0.17–0.82, *P* = 0.014) were still independently associated with HCC occurrence, whereas C4 and CIV did not reach statistical significance (C4: HR = 1.09, 95% CI 0.52–2.31, *P* = 0.82; CIV: HR = 0.52, 95% CI 0.16–1.73, *P* = 0.29; Table 5).

Taken together, the time-to-event analysis corroborates age and WBC as consistent predictors of HCC development in patients with biopsy-proven HBV-related cirrhosis.

### Calibration and assessments of the nomogram

**Figure 5:**
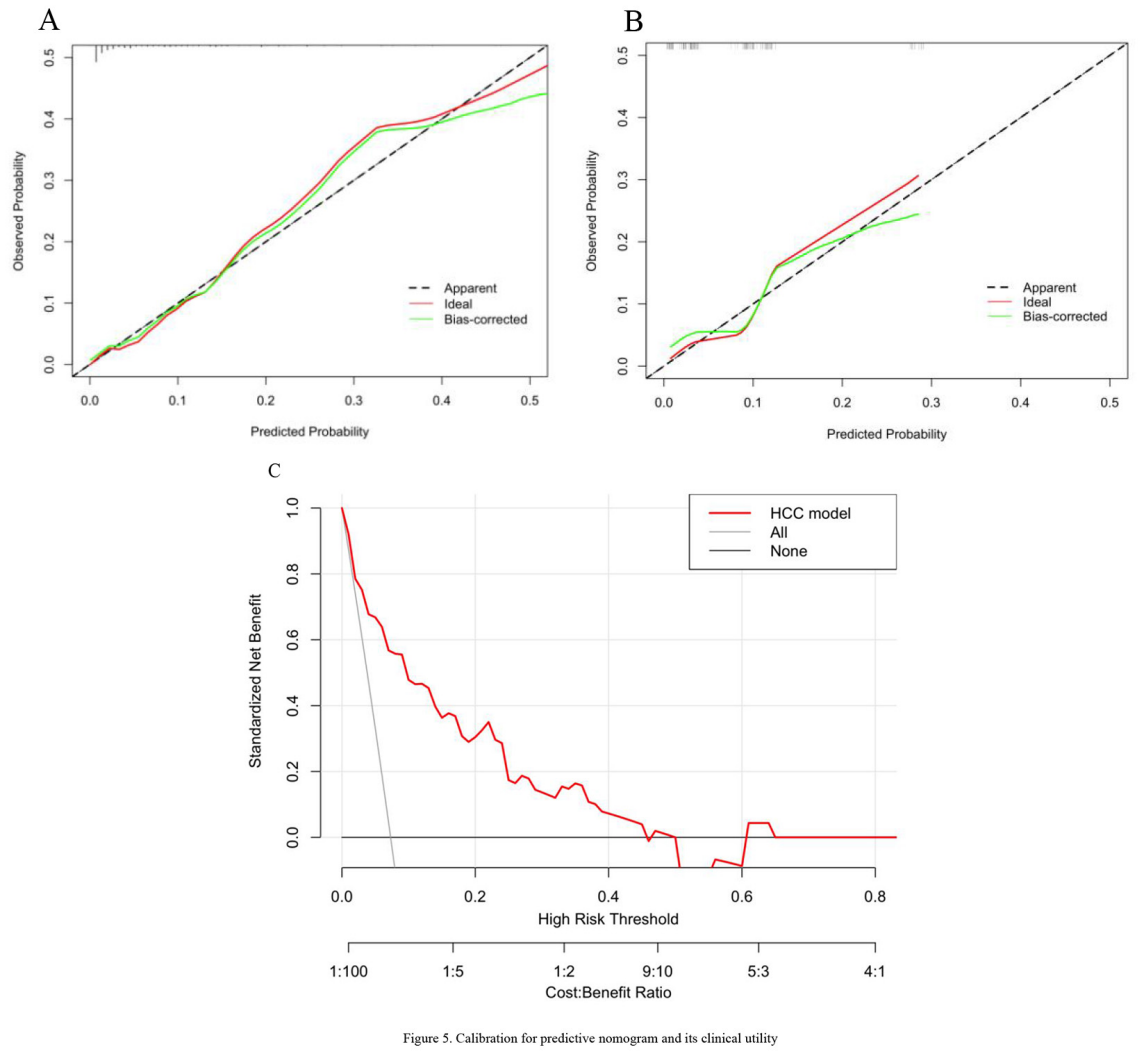
Calibration for predictive nomogram and its clinical utility. **(A, B)** Validity of the predictive performance of the nomogram in estimating the risk of HCC in the training cohort (*n* = 328) and validation cohort (*n* = 143). In the training set, the prediction curve showed perfect alignment with the dashed line. In the validation set, the prediction curve showed a wobbly feature likely resulting from a limited sample, but again did not drift much from the dashed line. **(C)** The clinical utility of the model was evaluated by Decision Curve Analysis (DCA). The results suggests that the model holds substantial value for clinical decision-making.

Calibration curves using the bootstrap method (1,000 times) were plotted (Figures 5A, B). The calibration slope and intercept were 1.02 and 0.03 in the training set, and 0.95 and 0.05 in the validation set, indicating good agreement. In the training set, the prediction curve showed perfect alignment with the dashed line, with an unadjusted C index of 0.869 (95% CI, 0.799–0.938) and a bootstrap-corrected C index of 0.869, suggesting agreement between the prediction and actual outcome. In the validation set, the prediction curve showed a wobbly feature likely resulting from a limited sample, but again did not drift much from the dashed line, with a C index of 0.762 (95% CI, 0.605–0.918) for the estimation of HCC risk.

Due to the limited number of HCC cases in this study, the model's predicted probabilities were predominantly concentrated in the low-risk range (< 0.5), resulting in a lack of data points in the high-risk range (>0.5) on the calibration curve. This is consistent with the epidemiological characteristics of HCC in patients with liver cirrhosis (33), but it may potentially affect the model's ability to identify high-risk individuals.

To further evaluate the clinical utility of the model, we performed Decision Curve Analysis (DCA) (Figure 5C). The results suggests that the model holds substantial value for clinical decision-making. The net benefit was superior to “treat-all” or “treat-none” strategies when the high-risk threshold was between 5 and 40%, supporting the use of this model to guide intensified surveillance (e.g., MRI instead of ultrasound, shorter follow-up intervals) in this risk range.

## Discussion

HCC predominantly arises in the context of liver cirrhosis, with approximately 80%−90% of cases developing against a background of established cirrhosis (27). While numerous predictive models, such as REACH-B (28), PAGE-B (29), and CAMD (34), have been developed to estimate HCC risk in patients with chronic hepatitis B, these models are primarily derived from heterogeneous cohorts that include patients across all fibrosis stages. A significant gap remains in the specific prediction of malignant transformation in patients with pathologically confirmed HBV-related cirrhosis. Our study aimed to address this unmet need by developing and validating a novel predictive model specifically for this high-risk population. The model, incorporating readily available clinical and serum biomarkers (age, WBC, C4, HA, and CIV), demonstrated satisfactory discriminative ability and calibration, offering a practical tool for risk stratification in clinical practice.

This study was grounded in the recognized pathological sequence of hepatocarcinogenesis. Nodular lesions in cirrhosis can evolve from regenerative nodules to premalignant lesions, early HCC, and eventually to typical HCC. Large cell changes (LCC) (35–37), irregular hepatocellular regeneration (IR) (38), and large regenerative nodules have been identified as morphological predictors of HCC in cirrhosis. Two large studies have demonstrated that LCC is a strong predictor of early HCC development, particularly in HBsAg-positive patients (36, 37). Therefore, we specifically included patients with a histological diagnosis of HBV-related cirrhosis at the S4 stage according to the Scheuer/Ludwig criteria (31).

The key predictors identified in our final model—Age, WBC, C4, HA, and CIV—are biologically plausible and align with the pathogenesis of HCC in cirrhosis. Advanced age is a well-established, non-modifiable risk factor for HCC across various liver diseases, including HBV-related cirrhosis (33, 39). The mean age of HCC patients in our training cohort was significantly higher than that of the non-HCC group (53.4 vs. 41.3 years), consistent with epidemiological trends in Asian populations. This likely reflects the cumulative effect of long-standing inflammation and genomic damage. The Kaplan–Meier survival curve also demonstrated a significant association between advanced age (dichotomized at the median) and the cumulative incidence of HCC (Log-rank test, *P* = 0.009). Furthermore, a univariable Cox proportional hazards analysis confirmed that older age was significantly associated with an increased risk of developing HCC [hazard ratio (HR) = 1.050, 95% CI 1.020–1.080, *P* < 0.001]. In a multivariable Cox model that included the key predictors from our nomogram, Age remained an independent risk factor (HR = 1.050, 95% CI 1.020–1.080, *P* < 0.001), and WBC (HR = 0.377, 95% CI 0.174–0.817, *P* = 0.014) also showed significant associations with HCC risk, consistent with the directionality and significance of our primary logistic regression model. In contrast, C4 (HR = 1.090, 95% CI 0.517–2.310, *P* = 0.817), and CIV (HR = 0.523, 95% CI 0.158–1.730, *P* = 0.287) failed to achieve significance in the Cox model. This discrepancy does not necessarily contradict our inclusion in the nomogram. C4 and CIV contribute incremental predictive gain mainly in the early fibrotic phase, whereas Cox coefficients average the hazard over the entire follow-up. Once Age and WBC are fixed, the remaining event number (*n* = 34) provides limited power to detect modest effects of C4 or CIV, leading to wide confidence intervals. Therefore, we consider C4 and CIV as supportive biomarkers that refine risk stratification in the nomogram, whereas age and WBC are the core drivers consistently significant in both static and dynamic analyses.

Notably, our model highlights the prognostic value of routine hematological and serological markers. A lower WBC count ( ≤ 4.5 × 10^9^/L) was independently associated with increased HCC risk. This finding may reflect the presence of hypersplenism in advanced cirrhosis, leading to leukopenia and a consequent state of relative immunosuppression. An immunosuppressive microenvironment is known to facilitate tumor immune evasion, providing a plausible mechanism by which leukopenia could contribute to hepatocarcinogenesis (40). Thus, WBC may serve as an indirect marker of impaired immune surveillance.

The protective effect of a higher C4 level (>0.15 g/L) in our model introduces an intriguing dimension to HCC risk assessment. The complement system plays a complex dual role in cancer, but our results suggest that adequate levels of C4, a key component of the classical complement pathway, might contribute to antitumor immunity in the liver microenvironment (41). A deficiency could impair complement-mediated clearance of pre-malignant cells, thereby increasing HCC risk. This finding warrants further investigation into the role of the complement system in HBV-related hepatocarcinogenesis.

The significance of HA and CIV underscores the critical link between active fibrogenesis and HCC risk. HA, secreted by activated HSCs, and CIV, a major component of the basement membrane, are direct indicators of ECM remodeling (42, 43). Elevated HA levels (>120 ng/ml) were associated with higher HCC risk, indicating ongoing active fibrogenesis. The integration of these fibrosis-specific markers provides a dynamic snapshot of the fibrotic milieu, reinforcing the concept of an “inflammation-fibrosis-carcinogenesis” axis. This finding suggests that serial monitoring of these markers could not only predict HCC risk but also gauge the response to antifibrotic therapies (43). Furthermore, the dynamic changes in WBC, potentially reflecting the inflammatory state, could also serve as a key monitoring indicator for antifibrotic treatment in patients with cirrhosis.

When positioned against established models like REACH-B (28) and PAGE-B (29), our model's novelty lies in its focus on a pathology-anchored cirrhosis-only cohort and the integration of fibrosis-specific serum markers. Existing models are invaluable for broad risk stratification in CHB patients but may lack precision in the distinct context of established cirrhosis. Therefore, our model is intended to complement these tools, offering a tailored risk assessment for the subgroup of patients with biopsy-proven S4 cirrhosis, in whom the absolute risk of HCC is highest.

Our study has several limitations that must be acknowledged. First, its single-center, retrospective nature and the relatively small number of HCC events, particularly in the validation set (*n* = 11), limit the generalizability and stability of the model. The event-per-variable (EPV) ratio was below the recommended threshold, with the EPV ratio being 6.8 in the training set, increasing the risk of overfitting (44). And we used complete-case analysis, which may introduce bias if data are not missing completely at random. Therefore, further validation using multicenter cohorts is needed. Second, we relied on a single baseline measurement of biomarkers; dynamic changes in these parameters over time likely hold greater predictive value. Third, our analysis did not account for competing risks, such as non-HCC related mortality. Fourth, we did not incorporate emerging predictors such as circulating tumor DNA or radiomic features, which could enhance predictive accuracy (45–48). Finally, our model was developed in a Chinese cohort with specific HBV genotype distributions (predominantly B and C) and epidemiological backgrounds. Therefore, its applicability to other ethnicities and global populations with different HBV genotypes and host factors may be limited. External validation in diverse international cohorts is necessary to assess its generalizability.

Despite these limitations, our study provides a clinically applicable tool for stratifying HCC risk in patients with HBV-related pathological cirrhosis. The identified biomarkers are routine, cost-effective, and provide insights into the underlying pathophysiological processes of immune dysfunction and fibrogenesis. Future directions should include external validation and the development of a more comprehensive prediction system by integrating multi-omics data, such as genomics and radiomics. Furthermore, based on risk stratification by this model, interventional studies could be designed to evaluate the efficacy of personalized surveillance strategies or preventive therapies in high-risk individuals.

## Data Availability

The raw data supporting the conclusions of this article will be made available by the authors, without undue reservation.
